# A comparative genomics study of carbohydrate/glucose metabolic genes: from fish to mammals

**DOI:** 10.1186/s12864-018-4647-4

**Published:** 2018-04-11

**Authors:** Yuru Zhang, Chaobin Qin, Liping Yang, Ronghua Lu, Xiaoyan Zhao, Guoxing Nie

**Affiliations:** 1 College of Fisheries, Henan Normal University, Xinxiang, 453007 People’s Republic of China; 2 College of Fisheries, Engineering Technology Research Center of Henan Province for Aquatic Animal Cultivation, Henan Normal University, Xinxiang, 453007 People’s Republic of China; 3 School of Computer and Information Engineering, Henan Normal University, Xinxiang, 453007 People’s Republic of China

**Keywords:** Carbohydrate/glucose metabolism, Comparative genomics, Zebrafish, Type 2 diabetes

## Abstract

**Background:**

Glucose plays a key role as an energy source in most mammals, but its importance in fish appears to be limited that so far seemed to belong to diabetic humans only. Several laboratories worldwide have made important efforts in order to better understand this strange phenotype observed in fish. However, the mechanism of carbohydrate/glucose metabolism is astonishingly complex. Why basal glycaemia is different between fish and mammals and how carbohydrate metabolism is different amongst organisms is largely uncharted territory. The utilization of comparative systems biology with model vertebrates to explore fish metabolism has become an essential approach to unravelling hidden in vivo mechanisms.

**Results:**

In this study, we first built a database containing 791, 593, 523, 666 and 698 carbohydrate/glucose metabolic genes from the genomes of *Danio rerio*, *Xenopus tropicalis*, *Gallus gallus, Mus musculus* and *Homo sapiens,* respectively, and most of these genes in our database are predicted to encode specific enzymes that play roles in defined reactions; over 57% of these genes are related to human type 2 diabetes. Then, we systematically compared these genes and found that more than 70% of the carbohydrate/glucose metabolic genes are conserved in the five species. Interestingly, there are 4 zebrafish-specific genes (*si:ch211-167b20.8, CABZ01043017.1, socs9* and *eif4e1c)* and 1 human-specific gene (*CALML6*) that may alter glucose utilization in their corresponding species. Interestingly, these 5 genes are all carbohydrate regulation factors, but the enzymes themselves are involved in insulin regulation pathways. Lastly, in order to facilitate the use of our data sets, we constructed a glucose metabolism database platform (http://101.200.43.1:10000/).

**Conclusions:**

This study provides the first systematic genomic insights into carbohydrate/glucose metabolism. After exhaustive analysis, we found that most metabolic genes are conserved in vertebrates. This work may resolve some of the complexities of carbohydrate/glucose metabolic heterogeneity amongst different vertebrates and may provide a reference for the treatment of diabetes and for applications in the aquaculture industry.

**Electronic supplementary material:**

The online version of this article (10.1186/s12864-018-4647-4) contains supplementary material, which is available to authorized users.

## Background

Carbohydrates are a ubiquitous fuel in biology. They are used as an energy source in most organisms, ranging from bacteria to humans. Increasing evidence suggests that high-carbohydrate diets are a potential risk factor for metabolic syndrome and type 2 diabetes (T2D) independent of energy intake [1–5]. However, the regulation of carbohydrate metabolism is complicated**.** In addition, unlike vigorous lipid metabolism research, large prospective studies that evaluate the relationship between carbohydrates and associated metabolic diseases have not been performed yet, and a systematic evaluation of high-sugar diets is still lacking. Consequently, there is an urgent need to analyze these areas comprehensively [6].

Existing studies have shown that the control and regulation of glucose homeostasis vary greatly between different organisms, leading to corresponding fluctuations in blood glucose levels across different classes of vertebrates. In these studies, researchers came to the realization that blood glucose concentrations among vertebrate groups are quite heterogeneous [7–9]. For example, in mammals, the blood glucose concentration is approximately 7 mM; birds have an almost 2-fold higher blood glucose concentration than mammals, and the concentrations in fish and amphibians are definitely lower than those in mammals [10]. In addition, glucose levels demonstrate intraspecies variability in fish, amphibians, reptiles, birds and mammals. While the control and regulation of carbohydrate/glucose homeostasis are currently well known in representative mammalian species (especially in rodents and humans), some of the dogmas created around these species may do not seem extend to other vertebrates. Glucose homeostasis in amphibians and reptiles has not received much attention. Thanks to the importance of fish in aquaculture [11–13], a significant number of studies have reported the control of glucose homeostasis in numerous fish species. Teleost fish are generally considered to be glucose intolerant. In fish, particularly in carnivorous species, prolonged postprandial hyperglycaemia is generally observed after feeding a carbohydrate-rich diet [8, 10]. Glucose regulation in fish has been discussed in numerous studies [12, 14–17]. Existing studies have shown that zebrafish have similar insulin dependence regulatory mechanisms as mammals. The genes and proteins responsible for the regulation of glucose metabolism, have been identified and demonstrated have similar regulation patterns and activity in zebrafish and mammalian [18–20]. There are also clear similarities and differences in body temperature, physiological, hormonal regulation, dietary carbohydrate use and so on when fish (at least *Oncorhynchus*) are compared with mammals. However, exploring divergence phenomena can be difficult. Currently, the physiological and molecular basis of this apparent glucose intolerance in fish is not fully understood. The reasons why basal glycaemia is different between fish and mammals and an understanding of the differences in carbohydrate metabolism amongst species are uncharted territory.

Over the past decade, molecular tools have been used to address some of the downstream components of carbohydrate/glucose metabolism and its regulatory processes, and these results have been used to better understand the roles played by carbohydrates and their regulatory paths. However, most results regarding a single gene or several related genes in single species tend to show strong sample biases and may not extrapolate from one species to another. Only genome-wide comparative approaches, which have the capacity to capture these multi-dimensional signals, can help achieve a systems-level understanding of the molecular underpinnings of carbohydrate metabolism [21, 22]. Nowadays, with the development of high-throughput sequencing technology and the availability of multiple, complete genomes of diverse life forms, comparative analysis can be used to provide a new qualitative perspective on homologous relationships between genes. This analysis, in turn, will enable a deeper understanding of the general trends in the evolution of genomic complexity and lineage-specific adaptations. Therefore, to confirm the complexity and heterogeneity of carbohydrate/glucose homeostasis, we adopted a comparative genomics approach in this study to compare all possible genes in carbohydrate/glucose metabolic pathway as a whole rather than analysing a single gene, pathway or species individually. We first constructed a database of every carbohydrate/glucose gene in *Danio rerio* (zebrafish), *Xenopus tropicalis* (frog), *Gallus gallus (chicken)*, *Mus musculus* (mouse) and *Homo sapiens* (human) and further annotated the functions of these genes in type 2 diabetes. Afterwards, we systematically compared these genes in the *Danio rerio*, *Xenopus tropicalis*, *Gallus gallus*, *Mus musculus* and *Homo sapiens* genomes. This study provides the first systematic genomic insights into carbohydrate/glucose metabolism, which may provide a reference for the prevention and therapy of human type 2 diabetes and may reduce aquaculture industry costs due to glucose intolerance.

## Results

### Carbohydrate metabolism gene mining from 21 KEGG pathways

In the KEGG database (http://www.genome.jp/kegg/), carbohydrate metabolism includes the following 15 pathways: glycolysis / gluconeogenesis [map00010], citrate cycle (TCA cycle) [map00020], pentose phosphate pathway [map00030], pentose and glucuronate interconversions [map00040], fructose and mannose metabolism [map00051], galactose metabolism [map00052], ascorbate and aldarate metabolism [map00053], starch and sucrose metabolism [map00500], amino sugar and nucleotide sugar metabolism [map00520], pyruvate metabolism [map00620], glyoxylate and dicarboxylate metabolism [map00630], propanoate metabolism [map00640], butanoate metabolism [map00650], C5-branched dibasic acid metabolism [map00660], and inositol phosphate metabolism [map00562]. It is known that carbohydrate metabolism homeostasis is regulated by many complex mechanisms in the digestive and endocrine systems. Thus, in order to garner a full list of carbohydrate/glucose-related genes, we also considered carbohydrate digestion and absorption [map04973], insulin secretion [map04911], insulin resistance [map04931], insulin signaling [map04910], glucagon signaling [map04922] and adipocytokine signaling [map04920] as target pathways that tightly regulate carbohydrate metabolism. Together, we gathered 545, 352, 418, 637 and 676 carbohydrate metabolism-related genes in zebrafish, frog, chicken, mouse and human, respectively, which were retrieved from the previously listed 21 target pathways in the KEGG pathway database (http://www.genome.ad.jp/kegg/pathway.html). Amongst these species, zebrafish lack information on insulin resistance [map04931], insulin signaling pathway [map04910], and adipocytokine signaling pathway [map04920] (http://www.genome.jp/kegg-bin/get_htext?dre00001); on the other hand, frog has no data on carbohydrate digestion and absorption [map04973], insulin secretion [map04911] and glucagon signaling pathway (http://www.genome.jp/kegg-bin/get_htext?htext=xla00001&filedir=%2fkegg%2fbrite%2fxla&length=).

**Fig. 1:**
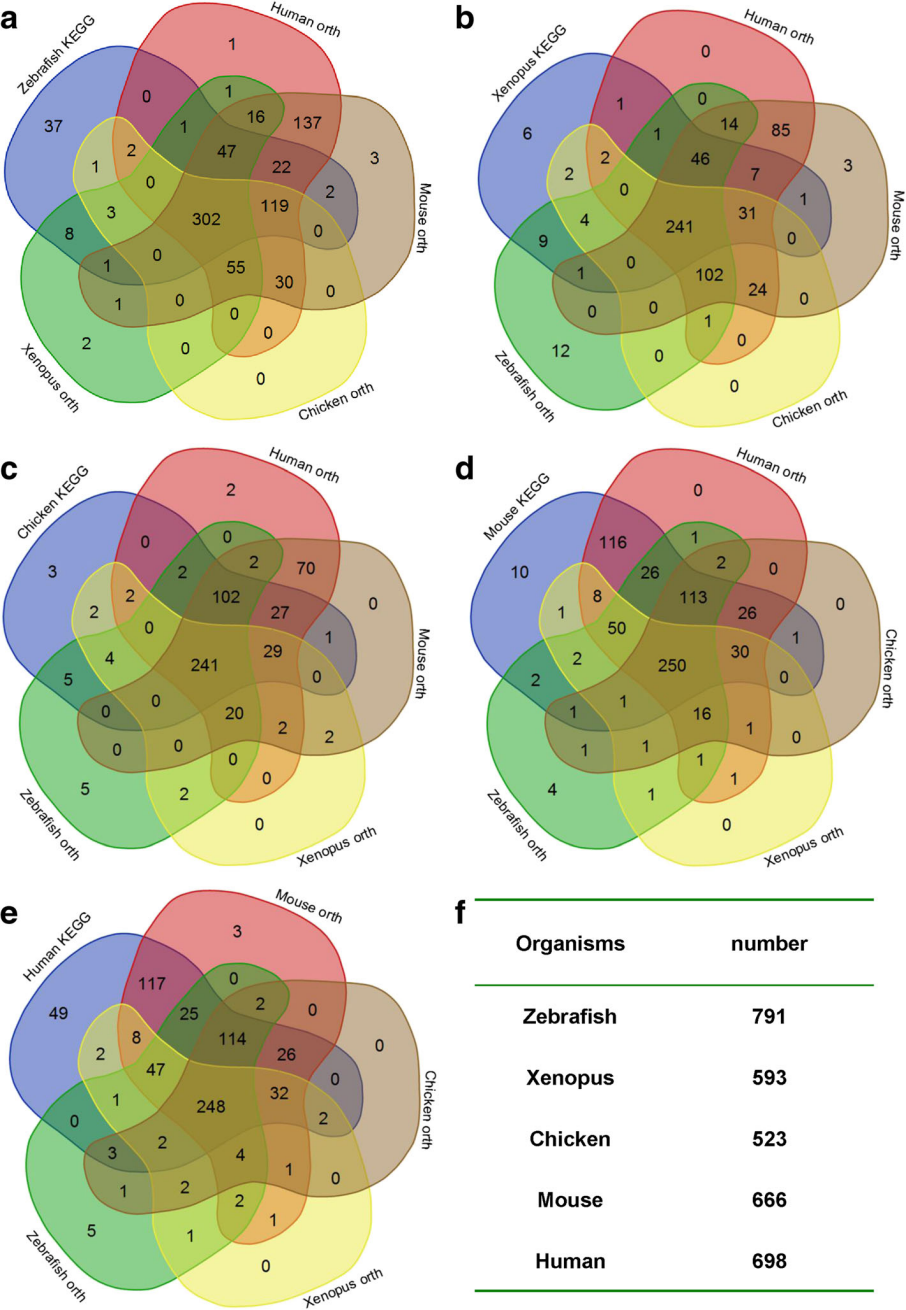
Carbohydrate/glucose metabolic genes in *Danio rerio* (zebrafish), *Xenopus tropicalis* (frog), *Gallus gallus* (chicken), *Mus musculus* (mouse) and *Homo sapiens* (human). These diagrams are modified from VENNY (see Methods). Numbers in the overlapping and non-overlapping areas of the diagram indicate the number of genes found by overlapping or unique sets of orthologues and KEGG genes in each species. **a** Venn diagram of 791 zebrafish carbohydrate/glucose metabolic genes, which are merged with 545 zebrafish KEGG database genes and human (676), mouse (637), chicken (418) and frog (352) KEGG resource gene orthologues. **b** Venn diagram of 593 frog carbohydrate/glucose metabolic genes, which are merged with 352 frog KEGG database genes and human (676), mouse (637), chicken (418) and zebrafish (545) KEGG resource gene orthologues. **c**-**e** Similar to the data mining in zebrafish and frog, there are 523, 666 and 698 carbohydrate/glucose genes in chicken, mouse and human, respectively. **f** The table gives an overall measure of how many genes were found in each organism. The gene list can be seen in Additional file 1: Table S1

To date, the most comprehensive lists of genes in the KEGG database has been collected for human and mouse, whereas the corresponding pathway genes in fish, bird and chicken are limited. Thus, based on the homology mapping in Ensembl Compara, we used 545, 352, 418, 637 and 676 carbohydrate genes in zebrafish, frog, chicken, mouse and human, respectively, as seed genes to search for orthologues across these species; then, we merged each organism’s KEGG gene lists with orthologues from the other four species. The overlapping genes in the five data sets were manually removed by the VennDiagram [23] package of R program; this process was performed online using the VENNY program (http://bioinformatics.psb.ugent.be/webtools/Venn/). Finally, we constructed five vertebrate carbohydrate/glucose gene databases containing 791, 593, 523, 666 and 698 genes in zebrafish, frog, chicken, mouse and human, respectively (Fig. 1 and Additional file 1: Table S1).

The biochemical functions of the 791, 593, 523, 666 and 698 carbohydrate/glucose metabolic genes in zebrafish, frog, chicken, mouse and human, respectively, were further annotated with Ensembl BioMart (http://www.ensembl.org/biomart/martview/199cd7da59587822b6e141a1afd51eed). Furthermore, when we chose zebrafish as a model organism, the functional annotation obtained from ClueGo [24] and DAVID [25] showed that 791 carbohydrate/glucose metabolic genes in zebrafish were not only involved in distinct carbohydrate metabolism processes but also played multiple roles in response to lipid metabolism, amino acid metabolism, drug metabolism, apoptosis and other biological processes (Additional file 2: Figure S1 and Additional file 3: Table S2).

Most of the 791 carbohydrate/glucose metabolic genes in this zebrafish database are predicted to encode specific enzymes that play roles in defined reactions, although experimental biochemical evidence for these conclusions may be lacking. Some genes that were predicted to encode the same or similar enzymes were classified into a superfamily/family. However, they may actually have the same function, distinct functions, or no biological function and may be involved in one or more reactions (Table 1). For example, 4 cytochrome b reductase (CYB5R) genes, which encode an essential enzyme that exists in soluble and membrane-bound isoforms, were present in zebrafish, each with specific functions [26]. The aldehyde dehydrogenase (ALDH) gene superfamily encodes enzymes that catalyse the oxidation (dehydrogenation) of aldehydes [27]. Within the zebrafish genome, 59 ALDH genes have been found. The ALDH gene products appear to be multifunctional proteins, possessing both catalytic and non-catalytic properties [28]. Only 15 of the 59 ALDHs may play a role in carbohydrate/glucose in our database. The solute carrier (SLC) groups of membrane transport proteins include over 300 members organized into 52 families [29]. In our database, the *slc2, slc5* and *slc37* gene family is involved in glucose transport, sodium/glucose cotransport and glucose-6-phosphate transport, respectively.

**Table 1:** Carbohydrate/glucose metabolic gene families in *Danio rerio*

Gene	Enzyme	Number in database
*hk*	hexokinase	3
*pfkA*	6-phosphofructokinase	6
*fbp*	fructose-1,6-bisphosphatase	7
*aldo*	fructose-bisphosphate aldolase	5
*pk*	pyruvate kinase	3
*ldh*	L-lactate dehydrogenase	4
*aldh*	aldehyde dehydrogenase	15
*g6pc*	glucose-6-phosphatase	4
*adpg*	ADP-dependent glucokinase	2
*pck*	phosphoenolpyruvate carboxykinase	2
*idh*	socitrate dehydrogenase	5
*ogdh*	oxoglutarate (alpha-ketoglutarate) dehydrogenase	3
*sdh*	succinate dehydrogenase	6
*pdh*	pyruvate dehydrogenase	4
*pgm*	phosphoglucomutase	4
*aldo*	fructose-bisphosphate	5
*ugt*	glucuronosyltransferase	11
*gmpp*	mannose-1-phosphate guanylyltransferase	3
*pfkfb*	6-phosphofructo-2-kinase	7
*ugp2*	UTP--glucose-1-phosphate uridylyltransferase	2
*b4galt1*	beta-1,4-galactosyltransferase	3
*pyg*	starch phosphorylase	4
*gys*	glycogen(starch) synthase	2
*chia*	chitinase	5
*cyb5r*	cytochrome-b5 reductase	4
*pdh*	pyruvate dehydrogenase	6
*mtm*	myotubularin-related protein	13
*plc*	phosphatidylinositol phospholipase	15
*slc*	solute carrier family	15

### Data mining and comparing of T2D-related genes reveal that most carbohydrate/glucose metabolic genes are involved in type 2 diabetes

**Fig. 2:**
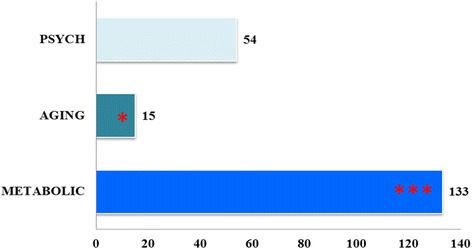
Disease annotation of 698 human carbohydrate/glucose metabolic genes using DAVID. The chart displays part of the significant enrichment analysis of disease in human carbohydrate/glucose metabolic gene database. The x-axis shows the gene number involved in the annotation terms. The y-axis represents the disease terms. One star denotes *P* < 0.05, whereas three stars denote *P* < 0.0001

An imbalance of major carbohydrate/glucose signaling pathways has previously been implicated in obesity, type 2 diabetes, non-alcoholic fatty liver disease, etc. [30, 31]. The human disease information annotation from DAVID shows that 133 of the 698 human carbohydrate/glucose genes are involved in metabolic disease, and some genes may be related to ageing and psychological disease. In addition, the top associated OMIM disease is noninsulin-dependent diabetes mellitus (Fig. 2 and Additional file 4: Table S3).

**Fig. 3:**
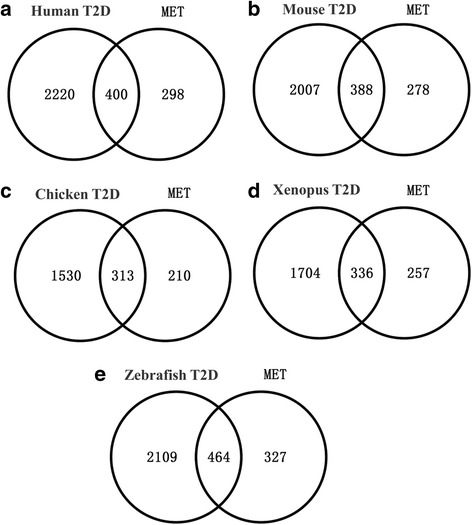
Venn diagram of the relationship between carbohydrate/glucose metabolic genes and type 2 diabetes-associated genes. **a** Comparison of 698 human carbohydrate/glucose metabolic genes with 2620 human T2D-related genes (400 of 698 carbohydrate/glucose metabolic genes overlapped with T2D genes). **b** Similar to the phenomenon in human genes, 388 of 666 mouse carbohydrate/glucose metabolic genes were related to T2D. **c** In chicken*,* 313 carbohydrate/glucose metabolic genes were associated with T2D. **d** In frog, 336 carbohydrate/glucose metabolic genes overlapped with 2024 T2D-related genes. **e** Similarly, 464 zebrafish carbohydrate/glucose metabolic genes were involved in T2D. Abbreviation: MET- carbohydrate/glucose metabolism. All genes are shown in Additional file 6: Table S5

To analyse the relationship between carbohydrate/glucose metabolic genes and human type 2 diabetes, human T2D-related genes were retrieved from the OMIM database (http://www.ncbi.nlm.nih.gov/omim/) using the keyword “type 2 diabetes”. A total of 681 MIM IDs, including genes, phenotypes, and loci, were downloaded from the OMIM database. Using Ensembl BioMart ID Mapping, we found 531 genes that were considered human T2D-related genes. To integrate the existing knowledge about T2D, we also collected information from T2D-Db, Type 2 Diabetes Genetic Association Database (T2DGADB) and T2D@ZJU to develop our T2D-related databases. To date, T2D-Db contains 330 manually curated candidate genes from PubMed literature and provides their corresponding information [32]. T2DGADB has collected 701 publications in T2D genetic association studies [33]. T2D@ZJU contains three levels of data associated with T2D, containing 2166 T2D genes [33]. By manually merging the genes mined from OMIM (531 genes), T2D-Db (330 genes), T2DGADB (701 genes) and T2D@ZJU (2166 genes) and excluding redundant genes with the online VENNY program, we identified 2620 additional human T2D genes in our database (Additional file 5: Table S4). Correspondingly, using the online Ensembl Compara tool, 2395, 1843, 2040, and 2573 orthologues of human T2D genes were found among mouse, chicken, frog, and zebrafish, respectively (Fig. 3 and Additional file 5: Table S4). Comparing T2D genes and carbohydrate/glucose metabolic genes, we found that 57.31% (400 of 698), 58.26% (388 of 666), 59.85% (313 of 523), 56.66% (336 of 593) and 58.66% (464 of 791) carbohydrate/glucose genes overlapped with the T2D genes of human, mouse, chicken, frog, and zebrafish, respectively (Fig. 3 and Additional file 6: Table S5).

### Systems bioinformatics comparison of glucose metabolic genes among zebrafish, frog, chicken, mouse and human

All 791 zebrafish carbohydrate/glucose metabolic genes were used to search for orthologues in the frog, chicken, mouse and human genomes using Ensembl BioMart (Methods) (Additional file 7: Table S6). Eventually, 709, 639, 738, and 738 carbohydrate metabolic genes were obtained in the frog, chicken, mouse and human genomes, respectively (Table 2). A comparison of these genes by the VENNY program revealed that over 80% of zebrafish carbohydrate/glucose genes have orthologues in frog, chicken, mouse and human. In addition, 596 zebrafish genes are conserved in all five organisms. Similarly, over 82%, 89%, 80% and 76% of frog, chicken, mouse and human carbohydrate/glucose metabolic genes, respectively, have orthologues in the other four species. Moreover, over 75% (zebrafish), 77% (frog), 84% (chicken), 71% (mouse) and 66% (human) carbohydrate/glucose metabolic genes are conserved in the five species, suggesting a high conservation of carbohydrate/glucose metabolism genes amongst vertebrates (Table 2). Interestingly, there are few species-specific genes that did not have orthologues in the other four organisms according to Ensemble Compara. Besides, most of these specific genes are assigned to an insulin-related pathway (Table 2 and Additional file 8: Table S7).

**Table 2:** Systems bioinformatics comparison of glucose metabolic genes by Ensembl BioMart

Organisms/statistic	Zebrafish (791) Orthologs	Frog (593) Orthologs	Chicken (523) Orthologs	Mouse (666) Orthologs	Human (698) Orthologs
Zebrafish	–	557(93.93%)	487(93.12%)	604(90.69%)	595(85.24%)
Xenopus	709(89.63%)	–	470(89.87%)	583(87.54%)	572 (81.95%)
Chicken	639 (80.78%)	489 (82.46%)	–	535 (80.33%)	532 (76.22%)
Mouse	738 (93.3%)	560 (94.44%)	504 (96.37%)	–	639 (91.55%)
Human	738 (93.3%)	562 (94.77%)	508 (97.13%)	649 (97.45%)	–
Conserved in five species	596 (75.35%)	460 (77.57%)	440 (84.13%)	473 (71.02%)	463 (66.33%)
specific	24 (3.03%)	4 (0.67%)	3 (0.57%)	6 (0.9%)	48 (6.88%)

### Manual assessment of the genome-specific genes

Orthologues are genes derived from a single ancestral gene in the last common ancestor of the compared species. Ensembl Compara is rated highly by publications analysing the performance of orthology prediction methods using both heuristic and phylogenetic methods to construct clusters and determine trees (Methods). However, a high degree of gene duplication, particularly in distantly related organisms, hinders orthologue identification, leading to false negatives. Thus, to avoid getting false species-specific genes that may play a key role in metabolic variance among different organisms and to further scrutinize the five species genome-specific genes identified by Ensembl Compara, we also used HomoloGene (https://www.ncbi.nlm.nih.gov/homologene) [34], TreeFam (Release 9, March 2013, 109 species, 15,736 families) (http://www.treefam.org/) and OrthoDB v9 (http://www.orthodb.org/) and assessed orthology based on RBH (reciprocally best hit, performing an NCBI BLASTP search using the default parameters). We also manually verified each genome-specific gene according to ZFIN (Zebrafish International Resource Center database, http://zfin.org/), MGI (Mouse Genome Informatics, http://www.informatics.jax.org/), Xenbase (Xenopus model organism database, http://www.xenbase.org/entry/), HGNC (HUGO Gene Nomenclature Committee, http://www.genenames.org/) and HCOP (HGNC Comparison of Orthology Predictions, http://www.genenames.org/cgi-bin/hcop). We found that some of the false-positive genome-specific genes account for gene duplication and alternative splicing (Additional file 8: Table S7). Lineage-specific gene duplications, which produce inparalogues, are likely to be a more common cause for violating the first assumption of Ensembl Compara detection. In other words, if one paralog evolves much faster than the other, this scenario could lead to a false negative under the Ensembl Compara method for orthologue detection (a pair of missed orthologues), but not to a false positive (no erroneous detection of orthologues). For example, in the zebrafish genome, ribosomal protein S6 kinase a 3a (*rps6ka3a*) has 2 transcripts (splice variants) and 11 paralogues (*rps6ka3b, rps6kal, rps6ka2, rps6ka1, rps6ka5, rps6kb1b, rps6kb1a, rps6ka4, sgk494a, stk32a* and *sgk494b*), whose sequence variation ranges from 24.27% to 86.47%. Paralogues with many sequence variations can obscure searches for orthologues. In addition, gene alleles that do not have homologue annotations in the Ensembl database are another reason for false negatives in human orthologue searches. For example, in the human genome, *ATF6B, RXRB, TNF* and *FLOT1* have 3, 5, 7 and 7 gene alleles, respectively, but only ENSG00000213676 (*ATF6B)*, ENSG00000204231 (*RXRB*), ENSG00000232810 (*TNF*) and ENSG00000137312 (*FLOT1)* have homologue information (Additional file 8: Table S7). Moreover, chitin is widely found in fungi and many invertebrate animals, but not in vertebrates [35, 36]; contrarily, some research data have indicated that chitin oligosaccharides are important for embryogenesis in vertebrates, whereas chitin synthase genes are present in numerous fishes and amphibians [37, 38]. In this study, chitinase was conserved in five species; however, chitin synthase was only found in the zebrafish and frog genomes (Additional file 8: Table S7).

**Fig. 4:**
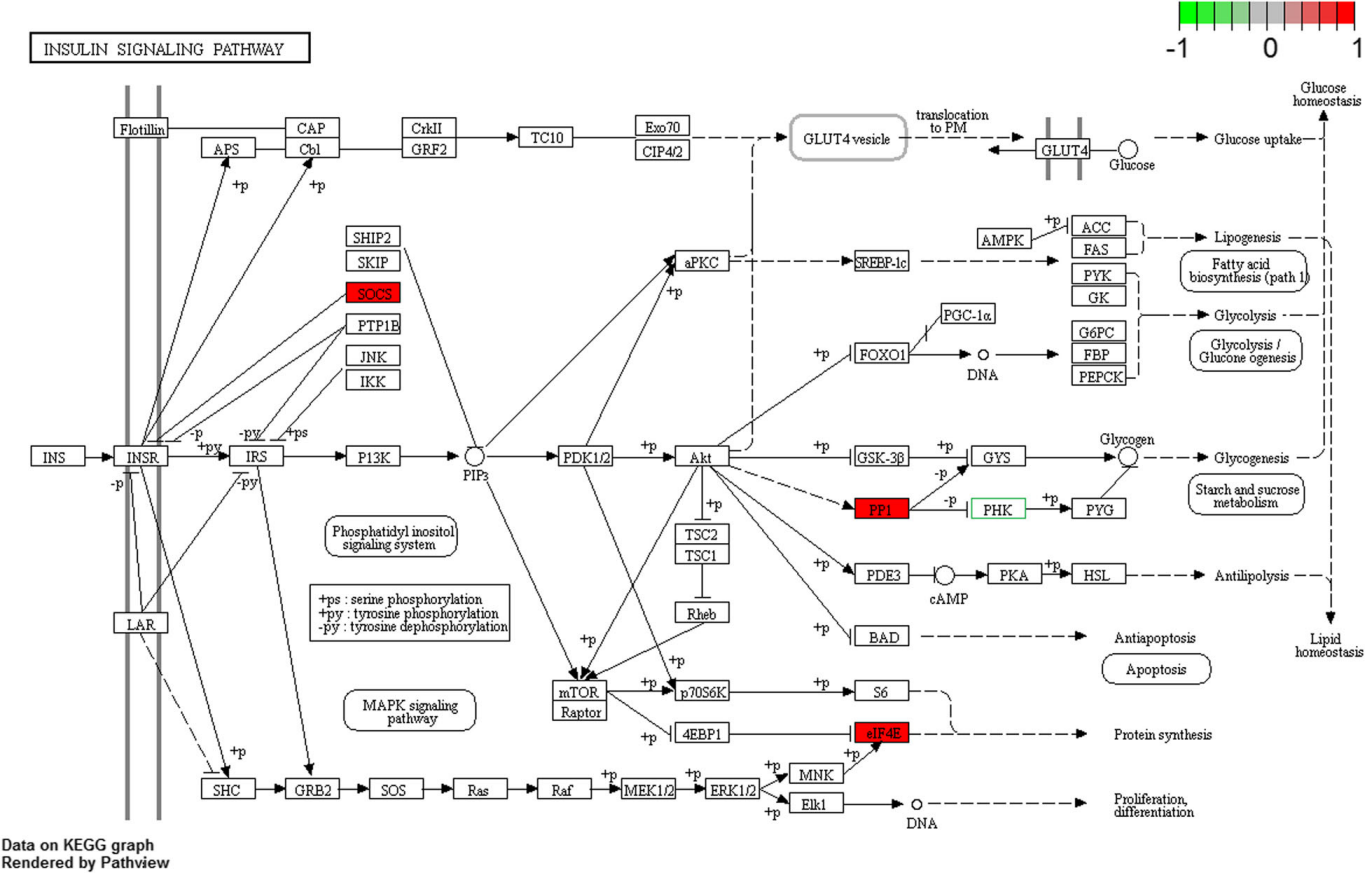
The location of *si:ch211-167b20.8, socs9, eif4e1c* and *CALML6* in the insulin signaling pathway. Zebrafish-specific *si:ch211-167b20.8* and human-specific *CALML6,* encoding PP1 and PHK, respectively, which are two proteins that play opposite roles in insulin pathways. Zebrafish-specific genes are shaded in red, human-specific CALML6 is shown in the green box

Notably, orthologues for 4 zebrafish-specific genes and 1 human-specific gene were not found in the other four organisms. In the zebrafish genome, orthologues for *si:ch211-167b20.8*, *CABZ01043017.1*, *socs9* (suppressor of cytokine signaling 9) and eif4e1c (eukaryotic translation initiation factor 4E family member 1c) were not found in the human, mouse, chicken and frog genomes (Table 3). In the human genome, *CALML6* (calmodulin like 6) has no orthologues in the other four genomes. *si:ch211-167b20.8* may encode protein phosphatase 1 (PP1) regulatory subunit 3A-like in the insulin resistance and insulin signaling pathways. This gene has 1 transcript (splice variant), 10 paralogues and 22 orthologues. According to sequencing similarities, *CABZ01043017.1* belongs to solute carrier family 2 (SLC2), which is a facilitated glucose transporter. Orthologues of *CABZ01043017.1* are mainly found in bony fish but not in other vertebrata. In the present study, a total of 120 SOCS genes from various species were globally investigated, of which *socs9* is a newly identified member in fish only [39] and *socs9* is a fish-specific duplication gene in fish. The *eif4e1c* gene is conserved in *S. cerevisiae*, *K. lactis*, *E. gossypii*, *S. pombe*, *M. oryzae*, *N. crassa, A. thaliana,* and *O. sativa* [40]. Interestingly, these four genes are found in bony fish but not in other vertebrata; they may have derived from the fish-specific genome duplication (FSGD) during the evolution of vertebrates [41, 42]. In the human genome, *CALML6* (calmodulin like 6), also known as *CAGLP* (calglandulin-like protein), may be a phosphorylase kinase (PHK) regulatory subunit containing conserved Ca^2+^ binding motifs, playing important roles in many biological processes [43]. This gene is conserved in chimpanzee, rhesus monkey, and dog. Regarding carbohydrate metabolic pathways, *si:ch211-167b20.8, CABZ01043017.1, socs9, eif4e1c and CALML6* participate in insulin resistance [PATH:dre04931], insulin signaling pathway [PATH:dre04910], adipocytokine signaling pathway [PATH:dre04920] and glucagon signaling pathway [PATH:hsa04922]. Moreover, we found that all of the genes are involved in the insulin signaling pathway except for *CABZ01043017.1* (Table 3 and Fig. 4). Pathway-based gene visualization was performing with the Pathview package [44] in R. This result confirmed that insulin plays a key role in the carbohydrate/glucose differences across species. In addition, in the insulin signaling pathway, PP1 (zebrafish-specific *si:ch211-167b20.8)* regulates glycogenesis through the inhibition of PHK (human specific *CALML6)*. These two genes variants may play a key role in the glucose metabolic differences between humans and fish. On other hand, *CABZ01043017.1* (solute carrier family 2) is involved in the insulin resistance and adipocytokine signaling pathways, which facilitate glucose transport and may also contribute to fish glucose intolerance.

**Table 3:** Zebrafish- and human-specific carbohydrate/glucose metabolic genes

Ensembl Gene ID	EntrezGene ID	*Associated Gene Name*	Paralogy number	Description	Pathway	Enzyme
Zebrafish specific						
ENSDARG00000097877	571,155	*si:ch211-167b20.8*	10	protein phosphatase 1 regulatory subunit 3A-like	04931 Insulin resistance [PATH:dre04931] 04910 Insulin signaling pathway [PATH:dre04910]	K07189 PPP1R3; protein phosphatase 1 regulatory subunit 3A/B/C/D/E
ENSDARG00000001937	100,334,818	*CABZ01043017.1*	12	solute carrier family 2	04931 Insulin resistance [PATH:dre04931]; 04920 Adipocytokine signaling pathway [PATH:dre04920]	K07299 SLC2A1; MFS transporter, SP family, solute carrier family 2 (facilitated glucose transporter), member 1
ENSDARG00000101985	100,003,691	*socs9*	14	suppressor of cytokine signaling 9	04910 Insulin signaling pathway [PATH:dre04910]	K04697 SOCS4; suppressor of cytokine signaling 4
ENSDARG00000012274	550,549	*eif4e1c*	6	eukaryotic translation initiation factor 4E family member 1c	04910 Insulin signaling pathway [PATH:dre04910]	K03259 EIF4E; translation initiation factor 4E
Human specific						
ENSG00000169885	163,688	*CALML6*	12	calmodulin like 6	04922 Glucagon signaling pathway [PATH:hsa04922]; 04910 Insulin signaling pathway [PATH:hsa04910]	K02183 CALM; calmodulin

### Glucose metabolism database platform construct

In order to facilitate the use of our data sets, we constructed a glucose metabolism database using JavaEE, Bootstrap3, MySQL 5.7 and Apache-tomcat-8.0.43. The glucose metabolism database (http://101.200.43.1:10000/) provides a user-friendly open platform with exhaustive search options for the retrieval of the information about the genes and their orthologues in human, mouse, frog, chicken and zebrafish. Users can download all reported information conveniently. The information available in the glucose metabolism database provides an integrated platform to better understand glucose intolerance in teleost fish and T2D in humans at a comparative genomics level.

## Discussion

**Fig. 5:**
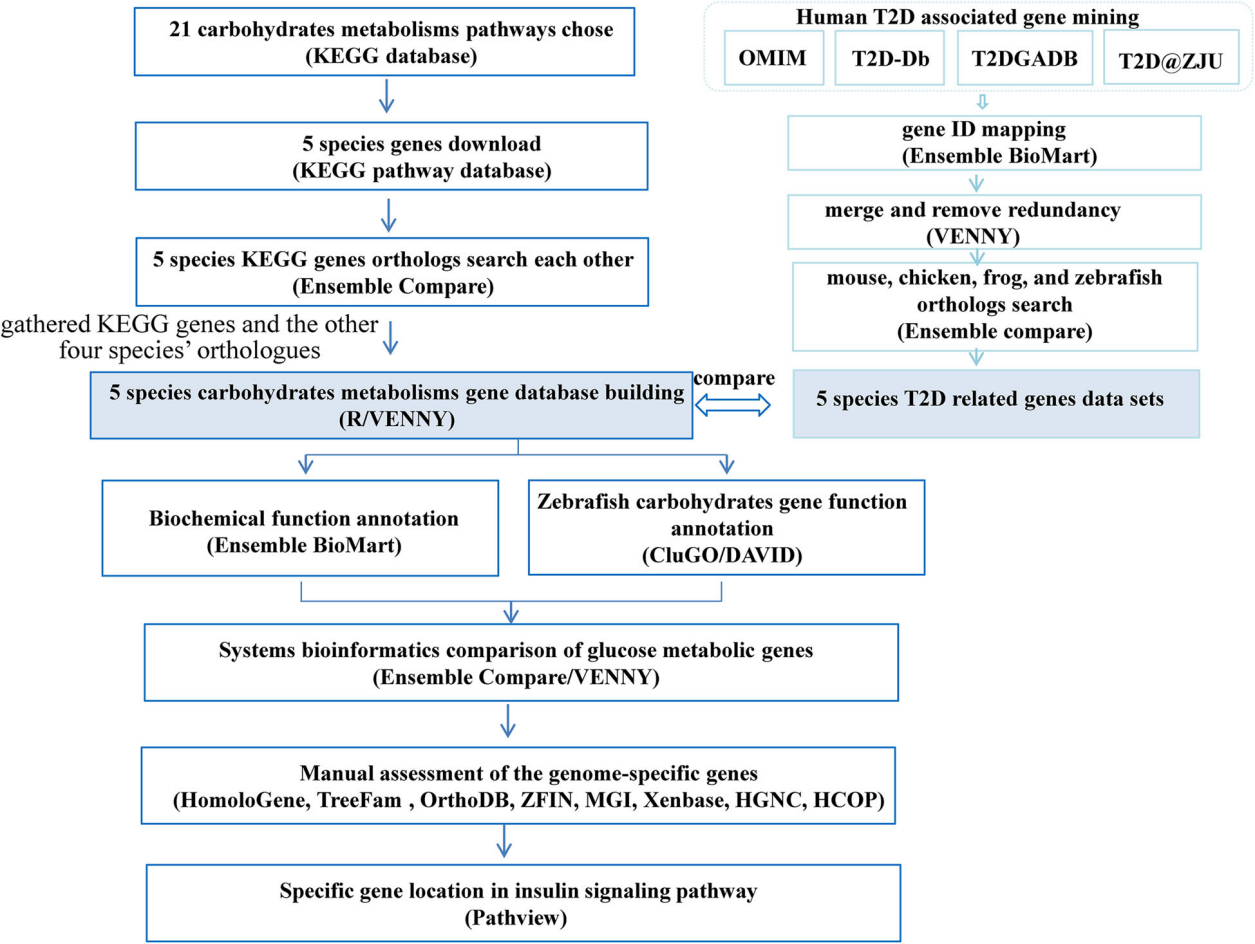
The study flowchart of this article. Methods are shown in brackets

Glucose plays a key role as an energy source in most mammals, but its importance in fish appears to be limited. Currently, the molecular basis for this apparent glucose metabolic fluctuation in different classes of vertebrates is not fully understood. Therefore, there is an urgent need for systems biology and integrative comparative genomics to delineate the information flow from metabolic enzymes to the pathway network. In this study, we chose *Danio rerio*, *Xenopus tropicalis*, *Gallus gallus*, *Mus musculus* and *Homo sapiens* as model organisms to comprehensively compare fish, frog, bird, mouse and human carbohydrate/glucose metabolic genes using comparative genomics (Fig. 5). To our knowledge, this is the first study to systematically analyse all the glucose genes, metabolic pathways, and their biological functions in these five animal models. Integrative carbohydrate/glucose metabolic comparative genomics will not only provide comprehensive knowledge about the biological functions of sugars and the mechanisms of health aquaculture; it will also expand the view of human type 2 diabetes and related metabolic diseases.

As shown in the workflow in Fig. 5, we first constructed a carbohydrate/glucose gene database of five vertebrates that contains 791, 593, 523, 666 and 698 genes in zebrafish, frog, chicken, mouse and human, respectively (Fig. 1 and Additional file 1: Table S1). These genes are involved in 15 carbohydrate metabolic pathways and 6 key regulatory pathways, including carbohydrate digestion and absorption [map04911], insulin secretion [map04911], insulin resistance [map04931], insulin signaling pathway [map04910], glucagon signaling pathway [map04922] and adipocytokine signaling pathway [map04920]. As such, this work provides useful information regarding carbohydrate metabolism for most enzymes in carbohydrate/glucose homeostasis. Although it may appear to be a daunting task to piece together the molecular components of glucose metabolism given the large amount of information across multiple organisms, the intrinsic relationships between different vertebrates can facilitate variance interpretation. Functional annotations show that these genes not only participate in carbohydrate metabolic processes but also play a key role in metabolic diseases, such as type 2 diabetes. By comparing the carbohydrate/glucose metabolic genes with those related to human type 2 diabetes, we found that over 57.31% of human carbohydrate metabolic gene are involved in T2D (Fig. 3 and Additional file 6: Table S5). This result further strengthens the case that carbohydrate metabolism plays a key role in metabolic disease.

Our comparison of the five organisms’ carbohydrate metabolic genes revealed that more than 66% of these genes are conserved in all five species. The results indicate that fish, frogs, chickens, mice and humans share high conservation in terms of carbohydrate genes and their roles in metabolic diseases, suggesting that *Danio rerio, Xenopus tropicalis, Gallus gallus* and *Mus musculus* are likely to be useful and insightful models for not only researching the fundamental biology of glucose metabolism but also exploring the mechanisms of human metabolic diseases. Remarkably, 4 zebrafish-specific (*si:ch211-167b20.8*, *CABZ01043017.1*, *socs9* and *eif4e1c*) genes and 1 human-specific (*CALML6)* gene did not have orthologues in the other four organisms. These genes are carbohydrate regulation factors, but their encoded enzyme are involved in insulin-related regulatory pathways. Interestingly, zebrafish-specific gene *si:ch211-167b20.8* (which encodes PP1) and human-specific gene *CALML6* (which encodes PHK) encode proteins that have opposite functions in insulin pathways (Fig. 4). In addition, fish-specific gene *CABZ01043017.1* appears to belong to the solute carriers 2A family (SLC2A, protein symbol GLUT) [45, 46], which are involved in the insulin resistance and adipocytokine signaling pathways; these proteins facilitate glucose transport and may contribute to fish glucose intolerance. This reaction was inferred from the corresponding reaction “SLC2A1-4 tetramers transport glucose from extracellular region to cytosol” in *Homo sapiens*. GLUT (glucose transporter) homotetramers associated with the plasma membrane mediate the facilitated diffusion of glucose from the extracellular space to the cytosol. The SLC2 family is comprised of a total of 14 members that transport different carbohydrates, most of which accept glucose [47]. Conserved sequence motifs in the GLUT proteins suggest the existence of shared structural features confirmed by in situ labelling and mutagenesis studies. Each GLUT protein has twelve membrane spanning domains organized to form an aqueous channel. While monomeric proteins can form such a channel and transport glucose, kinetic studies suggest that the functional form of the protein is a homotetramer. Different GLUT proteins are expressed in different tissues. Except for GLUT1, GLUT2, GLUT3 and GLUT4, other GLUT isoforms are less well characterized and can also transport other substrates, such as urate and myo-inositol [48–51].

In this study, we attempted to explain the differences in the glycaemic metabolism of different species using a genome comparison method. We found that most carbohydrate/glucose metabolic genes are conserved in human, mouse, chicken, frog and zebrafish; however, there are 4 zebrafish-specific genes and 1 human-specific gene, and 4 of these genes are involved in insulin pathways. Because of these gene distribution traits, we proposed that compared to conserved fundamental glucose metabolic enzymes, insulin association regulators may, in part, contribute more to variances in glucose metabolism between different species. For example, *si:ch211-167b20.8* has 10 paralogs (*ppp1r3* family genes), and studies have shown that the common variant of the 3′-untranslated region (UTR) in the corresponding gene (PPP1R3) was associated with insulin sensitivity [52–54]. Besides, all specific genes may have varying functions in different paralogs. For example, in the zebrafish genome, there are 6 paralogs of *eif4e1c*, which are *eif4e1a, eif4e1b, eif4eb, eif4e3, eif4e2rs1* and *eif4e2* according online Ensemble. This multiplicity of eIF4Es could reflect simple functional redundancy or it could indicate that eIF4E-related proteins support alternate roles. Robalino, J. et al. described two zebrafish eIF4E family members (*eIF4E-1A* and *eIF4E-1B*) that were differentially expressed and functionally divergent. Specifically, *eIF4E-1A* is a functional equivalent of human *eIF4E-1*. Surprisingly, although *eIF4E-1B* possesses all known residues thought to be required for interactions with ligands, it fails to interact with any of these components, suggesting that this protein serves a role other than that assigned to *eIF4E* [55]. However, the function of specific genes needs further experiments in all aspects of research. For example, research should be conducted to analyse the differences in insulin and glucose metabolism in model organisms or the hepatocyte cell line through RNAi, knockouts and other molecular techniques. Moreover, it is important to consider not only the enzyme and pathway conservation of a species but also its habitat preferences and functional complexity in comparisons of glucose metabolism between species [56], especially for species as divergent as fish and mammals. Understanding the inherited basis of glucose metabolism heterogeneity will require much more progress in identifying the mechanisms by which common, mostly non-coding variants influence metabolism. The combination of epigenetic measurements, genome editing, and high-throughput functional assays make it increasingly practical to characterize large numbers of gene variants and the processes that they affect. Biological insights gleaned from common and rare variant associations with carbohydrates will need to be integrated into a unified picture to fully understand the basis of carbohydrate metabolism and T2D.

## Conclusions

Glucose plays a key role as an energy source in most mammals, but its importance in fish appears to be limited. In this study, we chose *Danio rerio, Xenopus tropicalis, Gallus gallus, Mus musculus* and *Homo sapiens* as model organisms to comprehensively compare fish, frog, bird, mouse and human carbohydrate/glucose metabolic genes using comparative genomics. After an exhaustive analysis, we found that most metabolic genes are conserved in vertebrates. Our data and analysis may partly indicate that variances in carbohydrate utilization in vertebrates cannot be attributed to the genes that encode the most conserved fundamental glucose metabolic enzymes but to insulin association regulators and transport proteins. Our work may resolve some of the complexities of carbohydrate/glucose metabolic heterogeneity amongst different vertebrates and may provide a reference for the treatment of diabetes and for applications in the aquaculture industry.

## Methods

### Retrieval of carbohydrate/glucose metabolic genes and database construction

Most of the carbohydrate/glucose metabolic genes used in this study were initially downloaded from the KEGG pathway database (http://www.genome.ad.jp/kegg/pathway.html), which has to date collected 545, 352, 418, 637, and 676 carbohydrate/glucose metabolic genes from the zebrafish, frog, chicken, mouse and human genomes, respectively. Then, using frog, chicken, mouse and human KEGG genes (352, 418, 637, and 676, respectively), we searched for zebrafish orthologues with Ensembl BioMart and found 437, 512, 735 and 733 orthologous genes from frog, chicken, mouse, and human, respectively, in the zebrafish genome (Fig. 1a). We combined the KEGG genes and these orthologues genes, then discarded the overlapping genes, resulting in 791 carbohydrate/glucose metabolic genes were in the zebrafish genome. Similarly, using zebrafish (545), frog (352), chicken (418), mouse (637), and human (676) KEGG database carbohydrate/glucose metabolic genes, we searched the other four genomes for orthologues, then gathered each organism’s KEGG genes and the other four species’ orthologues for carbohydrate/glucose metabolic genes. Any overlapping genes were manually excluded with the VennDiagram [23] package of R program and using the VENNY online program (http://bioinformatics.psb.ugent.be/webtools/Venn/). After combining both sets of genes and discarding any overlapping genes in each organism, a total of 791, 593, 523, 666 and 698 carbohydrate/glucose metabolic genes were retrieved in zebrafish, frog, chicken, mouse and human, respectively (Fig. 1 and Additional file 1: Table S1). The BioMart Interface (http://www.ensembl.org/biomart/martview/199cd7da59587822b6e141a1afd51eed) was applied to standardize all gene IDs.

### Annotation of carbohydrate/glucose metabolic genes in zebrafish and human

The biological processes, KEGG pathway, disease, and molecular function annotation of the zebrafish (791) and human (698) genes were both processed through the ClueGo [24] plug-in of Cytoscape [57] and the DAVID 6.7 online database (https://david.ncifcrf.gov/) [25].

### Retrieval and comparison of T2D-associated genes in human, mouse, chicken, frog and zebrafish

Human T2D genes were gathered from the OMIM database (http://www.ncbi.nlm.nih.gov/omim/), T2D-Db [32], Type 2 Diabetes Genetic Association Database (T2DGADB) and T2D@ZJU [33]. First, we searched the OMIM database using the keywords “type2 diabetes” and downloaded 681 MIM IDs, including genes, phenotypes, and loci. Using Ensembl BioMart ID Mapping, we identified 531 genes that could considered human type 2 diabetes genes. We then downloaded genes from T2D-Db (330 genes), T2DGADB (701 genes) and T2D@ZJU (2166 genes). After merging and excluding redundant genes, 2620 additional human T2D genes were identified in our database (Additional file 5: Table S4).

Orthologue information on human T2D genes from the Ensembl (release 84) orthologue detection pipeline was obtained from Ensembl’s BioMart interface (http://www.ensembl.org/biomart/martview/199cd7da59587822b6e141a1afd51eed). Specifically, we retrieved Ensembl identifiers of orthologues to human genes and the identifiers of their translated protein products.

Briefly, gene families were identified from all sequences in the database by WU-Blastp and Smith–Waterman searches, followed by construction of a phylogenetic tree for each gene family to identify orthologous and paralogous relationships between gene pairs.

All orthologues of carbohydrate/glucose metabolism genes and T2D genes were then compared using VENNY, an interactive tool for comparing lists with Venn diagrams (http://bioinfogp.cnb.csic.es/tools/venny/index.html).

### False lineage-specific gene exclusion

Orthologues are typically identified based on the reciprocally best hits (RBHs) in both genomes using a basic local BLAST. However, a high degree of gene duplication, particularly in distantly related organisms, hinders orthologue identification by each method and leads to false organism-specific genes. To avoid getting false species-specific genes from Ensemble Compara, we also used HomoloGene (https://www.ncbi.nlm.nih.gov/homologene) [34], TreeFam (Release 9, March 2013, 109 species, 15,736 families) (http://www.treefam.org/), OrthoDB v9 (http://www.orthodb.org/) [58] and RBH (performing an NCBI BLASTP search, using default parameters) to detect orthology. We manually verified each genome-specific gene according ZFIN (Zebrafish International Resource Center database, http://zfin.org/), MGI (Mouse Genome Informatics, http://www.informatics.jax.org/), Xenbase (Xenopus model organism database, http://www.xenbase.org/entry/), HGNC (HUGO Gene Nomenclature Committee, http://www.genenames.org/) and HCOP: Orthology Predictions Search (http://www.genenames.org/cgi-bin/hcop).

### Database platform construct

Using JavaEE, Bootstrap3, MySQL 5.7 and Apache-tomcat-8.0.43, we constructed a glucose metabolism database.

## Additional files

Additional file 1: **Table S1.** Carbohydrate/glucose metabolic genes in 5 vertebrate organisms. All genes IDs were standardized as Ensembl gene IDs. Respectively, 791, 593, 523, 666 and 698 carbohydrate/glucose metabolic genes were present in the zebrafish, frog, chicken, mouse and human genomes. All gene descriptions were from Ensembl BioMart. (XLS 2875 kb)

Additional file 2: **Figure S1.** Biological process annotation of 791 zebrafish carbohydrate/glucose metabolic genes using ClueGO. The chart displays part of the significant enrichment analysis of Gene Ontology molecular functions in the zebrafish carbohydrate/glucose metabolic genes database. The x-axis represents the number of molecular function terms in Gene Ontology. One star denotes *P* < 0.05, whereas two stars denote *P* < 0.01. (PNG 106 kb)

Additional file 3: **Table S2.** KEGG pathway annotations of zebrafish carbohydrate/glucose metabolic genes by DAVID. We found 791 carbohydrate/glucose metabolic genes in zebrafish that were not only involved in distinct carbohydrate metabolic processes but also played multiple roles in response to lipid metabolism, amino acid metabolism, drug metabolism, apoptosis and other biological processes. (XLS 58 kb)

Additional file 4: **Table S3.** OMIM disease annotations of human carbohydrate/glucose metabolic genes by DAVID. Some genes are involved in noninsulin-dependent diabetes mellitus, thyroid carcinoma, paraganglioma and gastric stromal sarcoma, glycine encephalopathy, breast cancer and colorectal cancer. (XLS 25 kb)

Additional file 5 **Table S4.** Human T2D-related gene database and their corresponding orthologues in mouse, chicken, frog and zebrafish. To integrate existing knowledge about T2D, 2620 human T2D genes were identified in our database. Correspondingly, 2395, 1843, 2040, and 2573 orthologues of human T2D genes could be found among mouse, chicken, frog, and zebrafish, respectively. All genes IDs were standardized as Ensembl gene IDs. (XLS 464 kb)

Additional file 6: **Table S5.** The list of overlapping genes between carbohydrate/glucose metabolic genes and type 2 diabetes-associated genes. Respectively, 400, 388, 313, 336 and 464 human, mouse, chicken, frog and zebrafish carbohydrate/glucose metabolic genes are associated with T2D. (XLSX 30 kb)

Additional file 7: **Table S6.** Five species orthologues. Information including orthologue gene ID, gene name, % identity target species, identical genes to the query gene, orthology confidence and homology type are listed in the table. (XLSX 4387 kb)

Additional file 8: **Table S7.** Organism-specific genes by Ensemble Compara. Each gene was manually verified and annotated by HomoloGene, TreeFam, OrthoDB, ZFIN, MGI, Xenbase, and HCOP (Methods). (XLS 71 kb)

## Abbreviations

ALDH: Aldehyde dehydrogenase
CAGLP: Calglandulin-like protein
*CALML6*: Calmodulin like 6
CYB5R: Cytochrome b reductase
*eif4e1c*: Eukaryotic translation initiation factor 4E family member 1c
FSGD: Fish-specific genome duplication
GLUT: Glucose transporter
HCOP: HGNC Comparison of Orthology Predictions
HGNC: HUGO Gene Nomenclature Committee
MGI: Mouse Genome Informatics
PHK: Phosphorylase kinase
PP1: Protein phosphatase 1
RBH: reciprocally best hit
*rps6ka*: Ribosomal protein S6 kinase a
SLC: Solute carrier
*socs9*: Suppressor of cytokine signaling 9
T2D: Type 2 diabetes
T2DGADB: Type 2 Diabetes Genetic Association Database
TCA cycle: Citrate cycle
UTR: Untranslated region
Xenbase: Xenopus model organism database
ZFIN: Zebrafish International Resource Center database
