# Supplementary figures and images for: A comparative genomics study of carbohydrate/glucose metabolic genes: from fish to mammals
Source: BMC Genomics. 2018 Apr 11;19:246. doi: 10.1186/s12864-018-4647-4 (PMC5896114; doi:10.1186/s12864-018-4647-4)

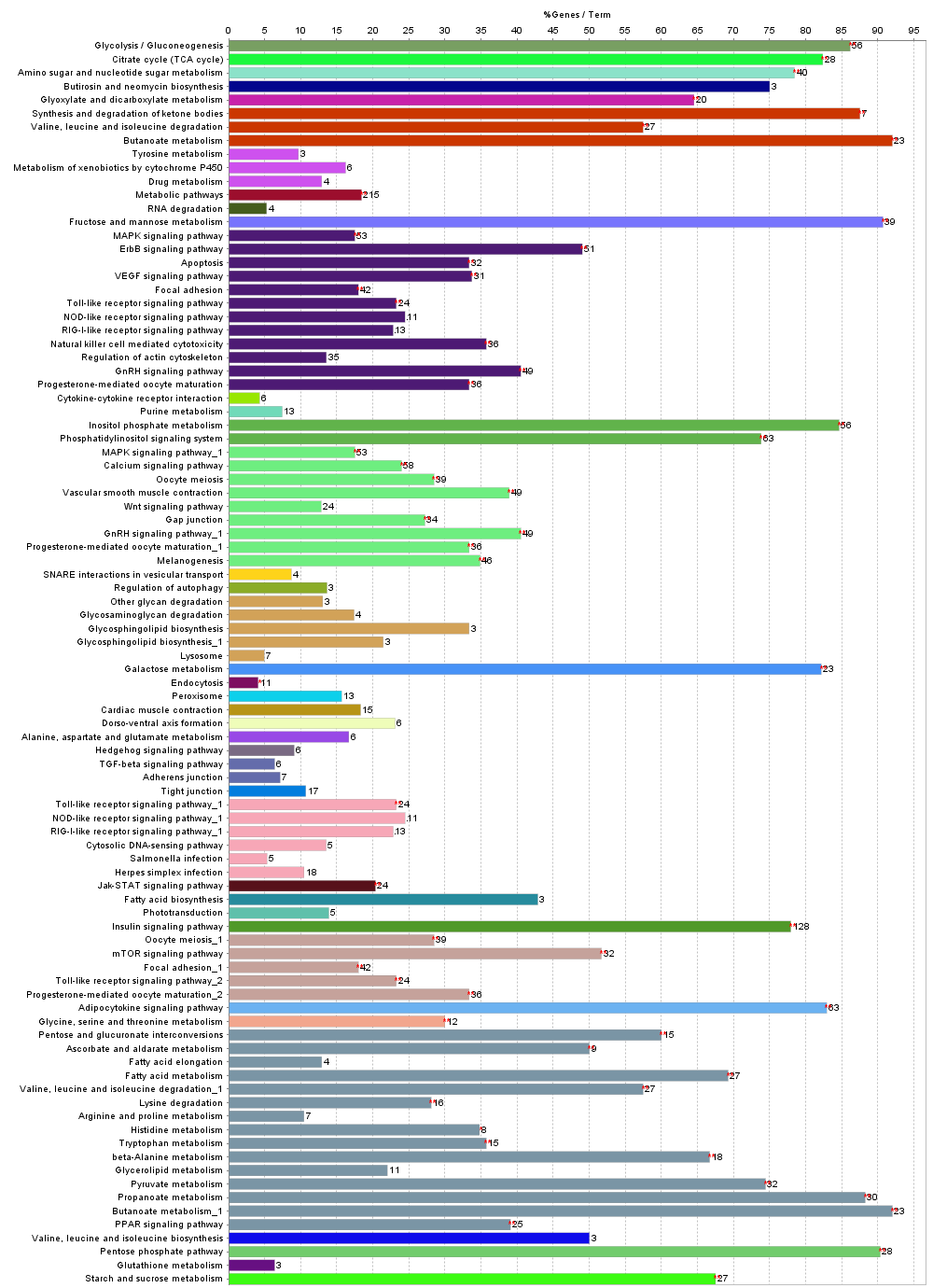

Supplement: Supplementary file 2 — Figure S1. Biological process annotation of 791 zebrafish carbohydrate/glucose metabolic genes using ClueGO. The chart displays part of the significant enrichment analysis of Gene Ontology molecular functions in the zebrafish carbohydrate/glucose metabolic genes database. The x-axis represents the number of molecular function terms in Gene Ontology. One star denotes P < 0.05, whereas two stars denote P < 0.01. (PNG 106 kb) [file 12864_2018_4647_MOESM2_ESM.png]
